# Chemist Eye: a visual language model-powered system for safety monitoring and robot decision-making in self-driving laboratories

**DOI:** 10.1039/d6dd00062b

**Published:** 2026-04-15

**Authors:** Francisco Munguia-Galeano, Zhengxue Zhou, Satheeshkumar Veeramani, Hatem Fakhruldeen, Louis Longley, Rob Clowes, Andrew I. Cooper

**Affiliations:** a Cooper Group, Department of Chemistry, University of Liverpool Liverpool UK aicooper@liverpool.ac.uk; b Midea Group Shanghai China; c Johnson & Johnson Toledo Spain

## Abstract

The use of robotics and automation in self-driving laboratories (SDLs) can introduce additional safety complexities, beyond those already present in conventional research laboratories. Personal protective equipment (PPE) is an essential requirement for ensuring the safety and well-being of workers in all laboratories, self-driving or otherwise. Fires are another important risk factor in chemical laboratories. In SDLs, fires that occur close to mobile robots, which use flammable lithium batteries, could have increased severity. Here, we present Chemist Eye, a distributed safety monitoring system designed to enhance situational awareness in SDLs. The system integrates multiple stations equipped with RGB, depth, and infrared cameras, designed to monitor incidents in SDLs. Chemist Eye is also designed to spot workers who have suffered a potential accident or medical emergency, PPE compliance and fire hazards. To do this, Chemist Eye uses decision-making driven by a vision-language model (VLM). Chemist Eye is designed for seamless integration, enabling real-time communication with robots. Based on the VLM recommendations, the system attempts to drive mobile robots away from potential fire locations, exits, or individuals not wearing PPE, and issues audible warnings where necessary. It also integrates with third-party messaging platforms to provide instant notifications to lab personnel. We tested Chemist Eye with real-world data from an SDL equipped with three mobile robots and found that the spotting of possible safety hazards and decision-making performances reached 88% and 95%, respectively.

## Abstract 2

Safety risks in self-driving laboratories (SDLs) are amplified by robotics and automation, motivating the development of real-time AI-driven monitoring and hazard-response systems.

## Graphical abstract

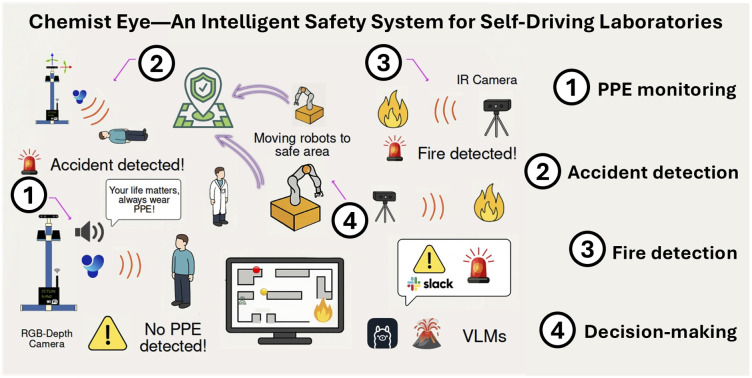

## 1 Introduction

Health and safety (H&S) is paramount in all workplaces, including offices, factories, laboratories, warehouses, manufacturing plants and healthcare facilities. The recent adoption of automated self-driving laboratories (SDLs) by the academic community^1–7^ raises some new H&S challenges in addition to the standard concerns for research laboratories, such as human–robot interaction (HRI) risks (*e.g.*, collisions), and possible fire and chemical hazards (*e.g.*, the potential for spills or contamination caused by robots^8,9^). Also, mobile robots are powered by lithium batteries that present an additional fire hazard. It is crucial to develop systems and protocols for SDLs that can deal with these risks.^10^ There are also opportunities to introduce new monitoring technologies into SDLs to manage more general laboratory hazards; for example, to monitor the proper use of PPE, which limits exposure to harmful liquids and solids, to identify possible accidents involving personnel, and to detect fires or likely sources of fires while improving awareness, control, and decision-making for both robots and lab users.

There are documented challenges regarding non-compliance with wearing PPE that are not specific to SDLs: the main causative factors are cognitive load and overfamiliarity.^11^ Cognitive load refers to the amount of mental effort used to process information and to carry out tasks and is particularly important for decision-making.^12,13^ Likewise, it has been recognized for decades that reliance on automation can lead to overfamiliarity and hence to PPE non-compliance.^14^ In principle, integrating new technologies in SDLs, such as robotics & automation (R&A), could lead to increased cognitive load, affecting the decision-making capabilities of individuals working in such environments. Furthermore, SDLs also impose an additional cognitive load on researchers who are less accustomed to chemical laboratories because SDLs often involve researchers from non-chemical fields, such as engineering or computer science, who may not have the same background in chemical safety. More generally, it is useful to explore new technologies for enforcing PPE compliance in research laboratories beyond SDLs.

One solution to counteract lack of PPE compliance is the use of verbal reminders as a means of persuasion, and indeed in well-run labs, colleagues are expected to do this. However, this assumes a scenario where there is more than one researcher present in the laboratory. To automate the enforcement of PPE compliance, or to detect accidents, we need reliable methods to trigger a corrective action, such as a warning. Several strategies in the literature focus on detecting PPE usage and accidents: these can involve wearable devices^15^ or vision-based methods.^16^ Such approaches have been applied mainly to construction sites, but there is a lack of comparable methodologies and systems that could be implemented in SDLs to provide feedback to workers and to modify robot behaviour.

Another risk in chemical laboratories is fire, where the most common causes are improper handling and storage of flammable chemicals, overheating during reactions, electrical faults in equipment, and static electricity.^17,18^ All laboratories have some form of a fire detection system, mostly using a combination of smoke detectors, heat sensors, and flame detectors.^19^ Upon detection, these systems trigger fire mitigation technologies such as gas-based suppression (CO_2_), powder-based (NH_4_PO_3_, K_2_CO_3_, KHCO_3_, Na_2_CO_3_, and NaHCO_3_), or fire sprinkler systems.^20^ Nevertheless, current fire detection systems in SDLs do not have any control over mobile robots used in automated workflows, which pose an increased risk due to their flammable lithium batteries. Moreover, such autonomous robots might continue to operate, irrespective of a fire or potential fire risk, unless a manual shutdown takes place.

**Fig. 1:**
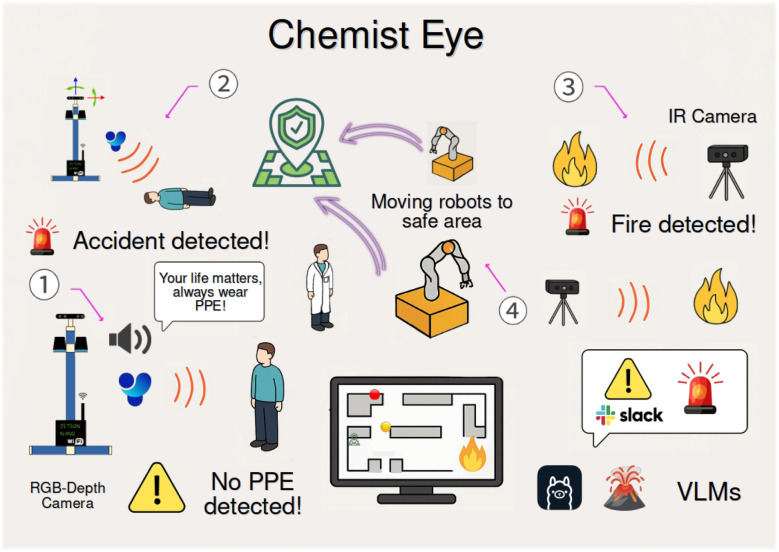
Chemist Eye overview. The system features four main capabilities: ① PPE compliance monitoring, ② accident detection, ③ fire detection, and ④ decision-making based on the identified issue.

In this paper, we introduce Chemist Eye (Fig. 1), a distributed safety monitoring system designed to improve situational awareness in SDLs. The system consists of monitoring stations equipped with RGB-Depth, and infrared (IR) cameras to observe the laboratory environment and to detect safety concerns. It runs in a Robot Operating System (ROS) environment, allowing communication and control of deployed mobile robots. It also integrates third-party messaging services to notify lab personnel in the event of potential problems. Additionally, Chemist Eye provides an interface for real-time monitoring of both lab robots and scientists. To facilitate detection of concerns and decision-making, the system integrates a Visual Language Model (VLM). These safety concerns include not wearing a lab coat, potential accidents (*e.g.*, a person lying prone on the floor), and fire detection. The system performance for spotting concerns under different conditions was tested and validated in simulation by using data from a real-life SDL at the University of Liverpool. Overall, our paper makes the following contributions:

• A distributed safety monitoring system for SDLs, featuring monitoring stations equipped with RGB, depth, and IR cameras, as well as speakers, to ensure safety by (i) monitoring PPE compliance, (ii) detecting possible accidents, and; (iii) identifying possible fire hazards.

• A methodology for leveraging cutting-edge technologies such as VLMs for the decision-making of robots operating in SDLs.

• A system that encourages workers to comply with PPE regulations employing automatic verbal reminders.

Beyond system integration, this work demonstrates that contextual prompting enables pre-trained VLMs to perform safety zero-shot reasoning in SDLs without task-specific training. This suggests a new approach towards rapidly deployable safety intelligence for autonomous research facilities.

### 1.1 Related work

In recent years, artificial intelligence (AI) tools, specifically vision-based methods, to detect PPE compliance have been investigated in fields ranging from health to construction. For example, Akib Protik *et al.*^21^ developed a system based on You Only Look Once (YOLO) to detect the use of face masks, a relevant problem during the COVID-19 pandemic. In another study,^22^ the authors develop three vision models based on YOLO, aiming to identify PPE compliance; more specifically, to try to determine in real-time whether a worker is wearing a hard hat, a vest, or both, using images and videos. More recent approaches also implement newer versions of YOLO for spotting PPE compliance among construction workers.^23^

Another reliable approach for detecting PPE compliance is by using sensors embedded in the PPE, such as radio frequency identification devices (RDIFs) and short-range transponders.^24^ For instance, Barro-Torres *et al.*^25^ present an approach to use the site's local area network (LAN) to communicate with RFIDs installed on PPE, which allows continuous monitoring of PPE compliance. Another example was reported in (ref. 26), where the authors demonstrate how to use AI to spot PPE compliance, emphasising protective glasses usage. Regarding systems that give feedback to workers when PPE non-compliance is detected, the approach presented by Gallo *et al.*^27^ implements a warning light that alerts workers after detecting that they are not wearing PPE.

For fire risks, besides the proven and reliable fire detection methods mentioned above (smoke detectors, heat sensors, and flame detectors), the scientific community has also developed AI-based methods for fire detection, such as applying AI to closed-circuit television (CCTV) systems. Vision-based fire detection systems can leverage existing CCTV infrastructure, such as in (ref. 28), where the authors used computer vision and deep learning techniques for early fire detection. As Pincott *et al.*^29^ explained, the traditional detectors mentioned above show several limitations during the ignition phase of a fire. For one thing, these systems can neither detect the location nor the size of the fire, which poses a limitation for decision-making. In the context of an SDL, it would be difficult to decide where to move the robots without knowing where the fire is—indeed, in the worst case, the robot could move into or through the fire, even if a predefined “safe parking” area is set.

In the context of chemistry and scientific discovery, recent work has explored the use of agent-based systems, focusing on tool-augmented reasoning, safety benchmarking, and risk-aware decision-making in predominantly text-based or computational settings.^30–34^

Notwithstanding valuable approaches in the literature, such as those mentioned above, there is still a gap regarding methodologies tailored to operate in SDLs. Moreover, contextual information positively impacts the decision-making^35,36^ and behaviour of agents and robots,^37^ helping them to adapt to the environment. Context gives significance to raw data by reducing ambiguity and directing attention towards a specific goal. Without contextual information, a situation may be challenging to interpret.^38^ In this work, we define context as the collection of conditions and circumstances linked to a particular environmental state (fires, accidents, and PPE compliance). The use of such information has the potential to enhance H&S in complex and challenging environments such as SDLs, where some robotic systems operate autonomously. Our paper aims to fill this gap with Chemist Eye, whose novelty lies in the use of cutting-edge AI tools such as VLMs and YOLO. In this way, we have sought to endow the system with useful contextual information, allowing it to leverage decision-making in SDLs by providing H&S capabilities for R&A systems operating under ROS, while providing verbal feedback to workers in real-time when needed.

## 2 Results

### 2.1 Chemist eye architecture overview

This section elaborates on the technical details of the components in Chemist Eye. Chemist Eye represents a multimodal safety architecture combining RGB-D perception, thermal sensing, and language-driven reasoning. In general, Chemist Eye seeks to provide the following core functionalities: (I) monitoring PPE compliance (focusing initially on lab coat usage); (II) monitoring workers' well-being status; (III) fire detection at pre-defined locations set by the user (*e.g.* a hotplate); (IV) decision-making for the robots operating in the lab based on (I), (II), and (III) and; (V) notification of potentially serious accidents through third party messaging services (*e.g.*, Slack).

**Fig. 2:**
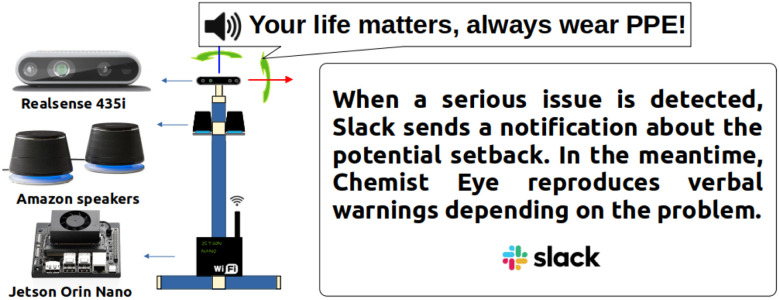
Chemist Eye RGB-D Station. The components that make up the Chemist Eye RGB-D Stations, include a Realsense 435i, two Amazon speakers and a Jetson Orin Nano mounted on an aluminium frame that allows adjustment of the camera.

**Fig. 3:**
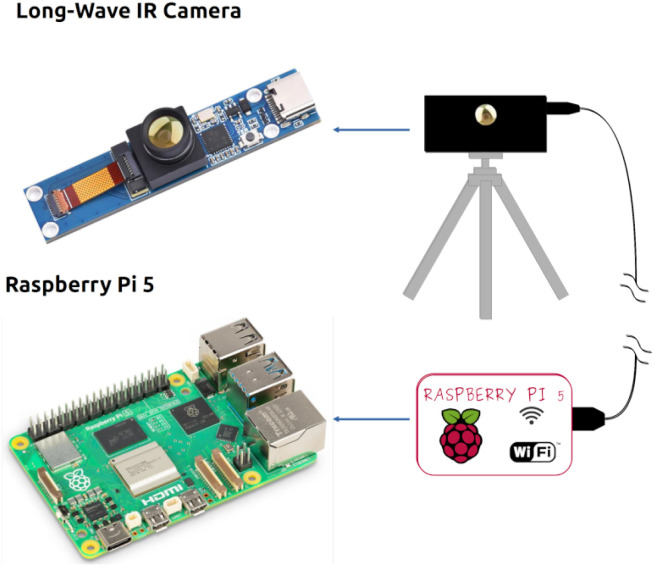
Chemist Eye Infrared (IR) Station. The components that make up the Chemist Eye Infrared (IR) Stations, include a long-wave IR Camera and a Raspberry Pi 5.

To implement such functionalities, the system integrates two types of vision stations, Chemist Eye RGB-D (Fig. 2) and Chemist Eye IR (Fig. 3). The Chemist Eye RGB-D Stations comprise a Jetson Orin Nano with Jetpack 5.1.3 as CPU, an Intel RealSense 435i, and two wired Amazon speakers that provide sound reproduction (audio messages to lab workers). All the components are fitted on an aluminium profile stand that allows the station to be levelled and the camera's view to be physically adjusted. The Chemist Eye IR Stations comprise a Raspberry Pi 5 running Raspbian OS as CPU and a long-wave IR camera mounted on a tripod that can also be mounted on a custom stand, with the aim of providing flexibility in terms of letting the user place the IR station in any convenient place, such as inside a fume hood or near a reaction station. The IR camera has an operating range from 20 °C to 400 °C, which represents a reasonable range for monitoring standard organic reactions. Hence, a temperature above 400 °C is abnormal and can be classified as a potential fire. Any desired threshold temperature can be set, and we used 55 °C in the experiments below as a test. For example, a lower threshold temperature could be used for detecting equipment that might be overheating, hence creating a possible fire risk.

**Fig. 4:**
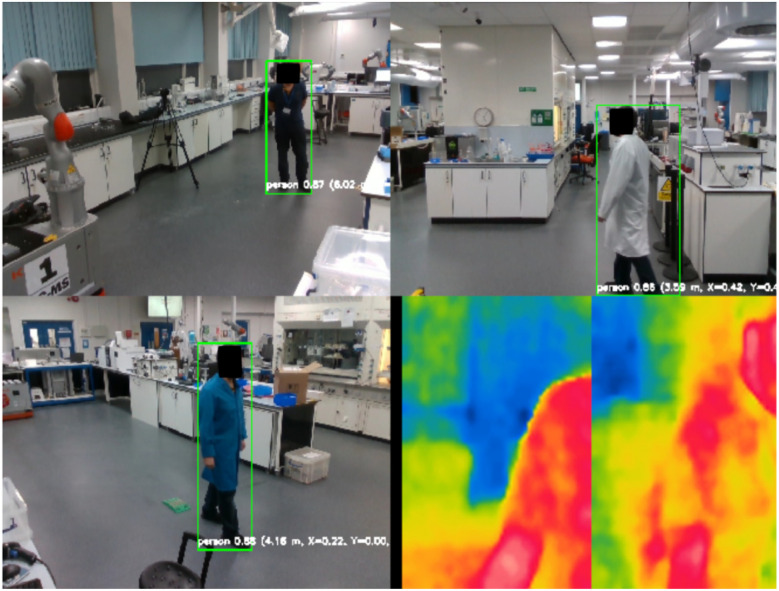
Illustration of the combined cameras' multiple workers view from both types of station (Chemist Eye RGB-D and Chemist Eye IR). You Only Look Once (YOLO) is used to spot people in the images, while the RealSense cameras are used to calculate their position with respect to the stations. Additionally, the IR camera streams can be seen at the bottom right corner of the figure.

**Fig. 5:**
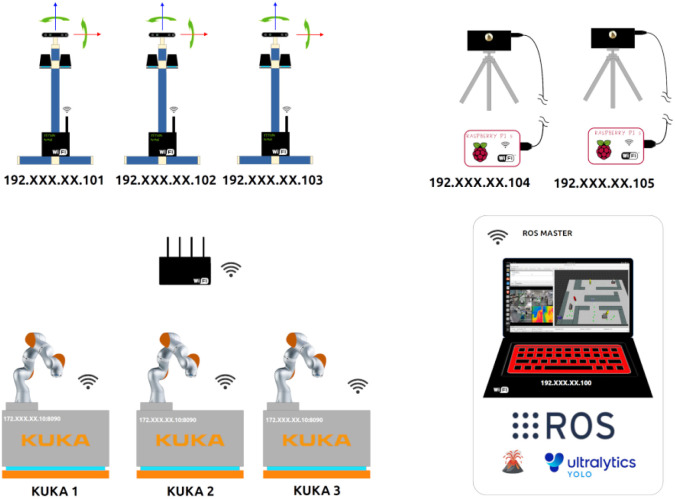
Network configuration of Chemist Eye. A central ROS Master communicates with the rest of the elements in the system through a Wi-fi router.

**Fig. 6:**
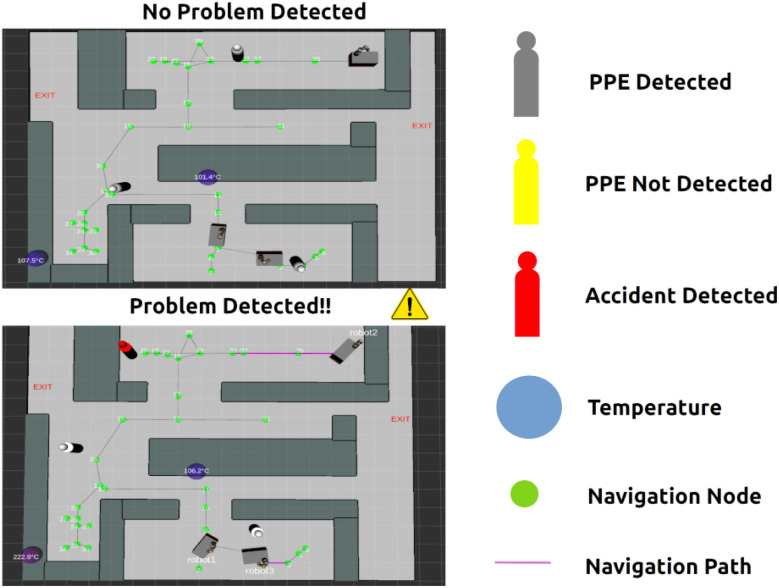
Map view produced by Chemist Eye. The virtual representation that RViz includes anonymised ”Meeples” (or pawns) representing the workers in the lab, along with their states (Personal Protective Equipment (PPE) detected = grey colour; PPE not detected = yellow; possible accident detected = red). Temperatures, at pre-defined locations, are captured by the Chemist Eye IR Stations; the blue spheres turn red when the temperature increases above a defined threshold, and this temperature is displayed above each sphere. The navigation nodes (green dots) depict the paths that the mobile robots can follow.

The system runs ROS, allowing data streaming from the Chemist Eye Stations (Fig. 4) and controlling robots connected to Chemist Eye, as depicted in Fig. 5. The system can integrate with ROS-compatible robots: in this study, we use KUKA KMR iiwa mobile robots. These robots follow a navigation path given by a set of nodes (green circles in Fig. 6).

When a contingency (accident) is detected, the system updates the robot path dynamically to reroute the robot. The PC that coordinates all the system's components also hosts the ROS master. At the same time, AI models like YOLO (Ultralytics) are used to locate people and their positions with respect to the Chemist Eye Stations by measuring distance with the RealSense cameras. Besides that, Chemist Eye supports several VLMs, more specifically LlaVA-7B and LlaVA-Phi3, which are used by Chemist Eye to query questions about live-stream images coming from the Chemist Eye Stations (Fig. 4).

When a worker is detected to be not wearing a lab coat, Chemist Eye reproduces verbal warnings such as: “Your life matters, always wear PPE!”, “Wearing PPE can save your life, wear it always”, or “PPE is your first line of protection, don't forget to wear it!”. Additionally, it switches the colour of the Meeple representing that individual to yellow (Fig. 6) and tries to restrict the robots from getting near the individual, aiming to safeguard the well-being of that worker by keeping away potential hazards, such as chemicals being transported by the robot. Once Chemist Eye detects that the individual is now wearing a lab coat, it stops reproducing the warnings and changes the colour of the Meeple to grey.

When Chemist Eye detects a potential worker accident or medical emergency through zero-shot classification *via* VLM, evaluating that the worker is no longer standing upright, it changes the Meeple's colour representing that worker to red (Fig. 6) and notifies other lab users through Slack about the potential accident. At the same time, Chemist Eye queries the VLM with the current view of the map and asks what are the best positions for the robot such that they do not pose a risk for that worker, with the aim of keeping the passage to the worker clear in case help is needed.

The information from the Chemist Eye IR Stations is used to detect possible fires, or precursors to fires, by determining if one of these stations measures a temperature that exceeds a specific threshold. In this study 55 °C was selected as the threshold. This temperature is low enough to be safely achieved in the laboratory using standard hot-plates, while still sufficiently above both body and ambient temperatures, avoiding false triggers from human presence. The system will query the VLM by feeding the image of the current laboratory map and asking what the best locations are to keep the robots away from the potential fire. The VLM then returns the node numbers, and Chemist Eye sends the robots to that location. After this, Chemist Eye sends a Slack message to other laboratory users so that they can evaluate the situation and take appropriate measures. It would be straightforward to connect this system in the future to a visible and audible alarm, or to link it into existing conventional fire detection systems.

All Chemist Eye components communicate over a network using fixed IP addresses, and a ROS Master Node coordinates the system. Hence, Robot Visualizer (RViz) is used to stream a map representation and markers, such as anonymised Meeples, for the individuals detected by the cameras, temperature indicators, and robot URDF models (Fig. 6). This map view can be attached to a warning message in Slack and can provide helpful information about where an accident has happened so that co-workers or emergency personnel can leave the area by the safest available route, while maintaining privacy and not sharing or keeping images of the actual accident. The view of the map can be streamed, and in this way, Chemist Eye features a user-friendly interface for real-time monitoring of SDLs. Chemist Eye addresses privacy concerns by not storing any raw camera images during normal operation. The system, instead, leverages zero-shot capabilities of the VLM to make decisions, hence eliminating the need to create or store datasets. Moreover, the VLM runs offline, which adds another layer of privacy since no images are stored either locally or shared online, whether or not they are used to detect PPE compliance or a worker accident. When the system shares an image through Slack, the image is a map where individuals are anonymised markers; however, if the user decides to use a cloud-based LLM service, then how the data is handled becomes subject to the provider's privacy policies. When multiple hazards are detected simultaneously, Chemist Eye applies a priority hierarchy reflecting laboratory risk levels: fire hazards are treated as the highest priority, followed by potential medical emergencies, and finally, PPE non-compliance. This ordering mirrors standard laboratory safety protocols. We note that General Data Protection Regulation (GDPR) laws may influence the adoption of such approaches in some countries.

In terms of system robustness and fault tolerance, Chemist Eye features several mechanisms designed to improve reliability. For example, each station, including both types (RBG-D and IR) run a lightweight API service, which, in case of any fault, would restart and continue operating. Moreover, the distributed station architecture of Chemist Eye endows the system with partial redundancy; in other words, while one station is down, the rest can still operate normally.

### 2.2 Methods

**Fig. 7:**
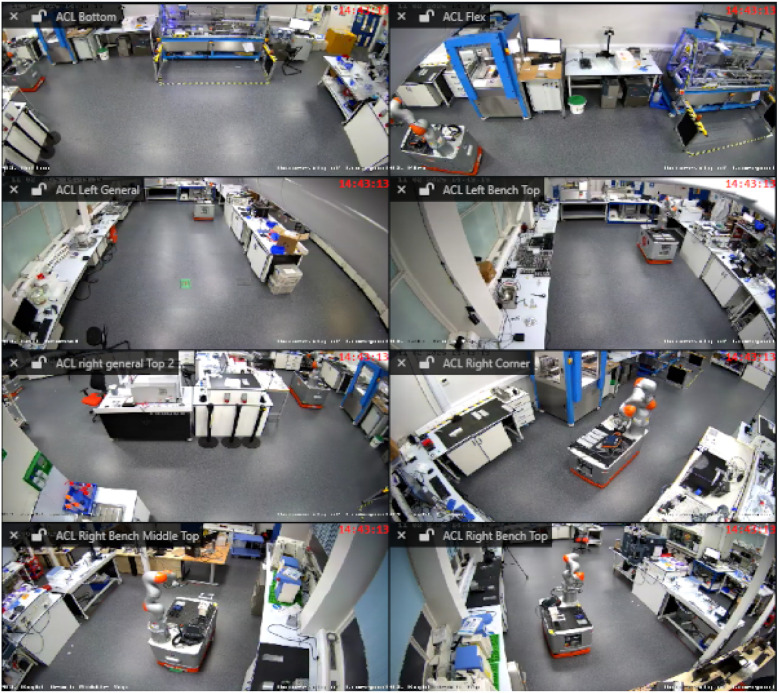
CCTV views of the Autonomous Chemistry Laboratory (ACL) at The University of Liverpool. This shows the overall lab set-up; specific camera stations were used to collect data for Chemist Eye.

This section describes the experimental setup, data generation process, and evaluation procedures used to validate this first version of Chemist Eye. The experiments were conducted in the Automation Chemistry Lab (ACL). The ACL, shown in Fig. 7, is equipped with three KUKA mobile robots and various labware, including a Powder X-ray Diffraction (PXRD), Nuclear Magnetic Resonance (NMR) and Liquid Chromatography-Mass Spectrometry (LCMS) machines, as well as hot plates, ultrasound baths, syringe pumps and solid dispensers. Several fully automated workflows have been implemented in the ACL,^4,5,7^ making it a suitable environment for validating Chemist Eye. The experiments were conducted within the ACL, whose layout could not be modified due to ongoing research activity. Rather than optimising for multiple hypothetical environments, we focused on demonstrating infrastructure-aware safety monitoring within a realistic laboratory configuration. Because Chemist Eye interfaces directly with ROS-based mapping and navigation stacks, the system is inherently transferable to other laboratory spaces without requiring architectural redesign.

We validated the system across six Case Studies in simulation using real-world data collected from the ACL and saved in bag files, allowing real-time reproduction of the laboratory events, thereby facilitating the evaluation of Chemist Eye while ensuring a safe benchmarking by not risking either equipment or personnel. For all experiments, we evaluated the performance of two VLMs: LlaVA-7B and LLaVA-Phi3, and a commercial LLM for the last case study (GPT-4o mini). Hazard locations and simulated events were randomised to reduce positional bias and ensure that the models were evaluated in diverse spatial configurations.

To generate the datasets used in the evaluation, we first recorded several video streams in bag files from the three RGB-D stations. Using a Python script, we then extracted images from the bag files, introducing random pauses between captures to avoid saving images showing similar poses. For the first two case studies, we sampled 400 images and manually divided them into four categories: PPE, NOT_PPE, PRONE, and NOT_PRONE, aiming to test the performance of several prompts and the zero-shot capabilities of the system. For the final case study, we sampled 900 additional images depicting one, two, or three individuals in scenarios where one or more participants might wear a lab coat or simulate a prone position, while others stand normally. These 900 images were categorised into three classes: NORMAL, NOT_PPE, and PRONE. Images in the NORMAL class serve as a common baseline, allowing benchmarking of the system in detecting PPE or simulating worker accidents, while reducing the effort required to produce additional images.

For safety/ethical reasons, risky scenarios such as intentional overheating equipment or actual smoke or fire generation within a laboratory setting were not conducted physically. Likewise, we did not instruct personnel to simulate medical emergencies in proximity to autonomous robots. Such experiments would not comply with institutional safety regulations. Hence, we decided to adopt a controlled validation approach, which is based on real-world data replay from ROS bag files, which also allows the real-time reproduction of events. This approach allows the real-time reproduction of events while allowing reproducible evaluation of hazardous scenarios without endangering workers or infrastructure. This approach is a de facto practice in robotics and autonomous systems research, where simulation is routinely employed for safety-critical testing prior to real-world deployment.

### 2.3 Case studies

#### 2.3.1 Case study 1: PPE compliance detection

The first experiment evaluates the accuracy of Chemist Eye in detecting PPE compliance. Multiple video recordings of a lab worker both wearing and not wearing PPE were captured using Chemist Eye RGB-D stations and stored in ROS bag files. From these recordings, the collected images were manually categorised into two classes: wearing a lab coat and not wearing a lab coat. Although the dataset size is modest, the objective of this study was not to train a perception model but to evaluate zero-shot capabilities of pre-trained VLMs within a safety pipeline. In other words, the objective was to assess how accurately VLMs can detect PPE compliance without requiring any training or fine-tuning. We evaluated the VLMs using a series of queries (*Q*_1_–*Q*_4_) described in Table 1. Keyword-based decision-making (*e.g.*, in *Q*_3_ and *Q*_4_) was motivated by an analysis of the VLM responses, where certain terms (such as LAB COAT and WHITE) appeared frequently, with earlier terms in the list being more common. Some queries also involved combinations of multiple prompts. Performance was measured using accuracy and the rate of hallucinations, which we defined as instances where the VLMs failed to follow the query format or returned unrelated content.

**Table 1:** Summary of queries used to assess lab coat compliance (*Q*_1_–*Q*_4_) and accident detection (*Q*_5_–*Q*_10_). Queries consist of single or sequential prompts, and responses were interpreted *via* keyword rules. Hallucinations are defined as instances where the VLM failed to follow the query format (*e.g.*, not replying YES/NO when instructed)

Query	Prompt(s)
*Q*_1_	Is the person wearing a lab coat? ONLY reply with YES or NO
*Q*_2_	Is the person wearing a WHITE lab coat? ONLY reply with YES or NO
*Q*_3_	What is the person wearing? Keywords: WHITE, LAB COAT, COAT ⇒ PPE present
*Q*_4_	Is the person wearing a lab coat? Is the person wearing a white lab coat? What is the person wearing? Decision based on keywords: WHITE, LAB COAT, COAT
*Q*_5_	Is the person prone? ONLY reply with YES or NO
*Q*_6_	Is the person LYING on the floor or KNEELING or SITTING or CROUCHING or BENDING OVER or SQUATTING DOWN? ONLY reply with YES or NO
*Q*_7_	Is the person standing? ONLY reply with YES or NO
*Q*_8_	Is the person standing or walking? ONLY reply with YES or NO
*Q*_9_	What is the person doing? If answer contains: KNEELING, SITTING, CROUCHING, BENDING, SQUATTING, LYING ⇒ prone. WALKING, STANDING, CHECKING, EXAMINING, LOOKING, WORKING ⇒ not prone
*Q*_10_	Is the person standing? ONLY reply with YES or NO. Is the person walking? ONLY reply with YES or NO. What is the person doing? Keywords interpreted as in *Q*_9_; fallback used when prior answers are ambiguous

This experiment focused on evaluating the performance of the two VLMs in analysing the video streams from the Chemist Eye RGB-D stations. All the models were evaluated based on their ability to correctly classify workers as either wearing or not wearing a lab coat. The performance metrics used were accuracy rate, hallucination rate, and time. Table 2 summarises the results. Both VLMs demonstrate superior performance for *Q*_3_ and *Q*_4_, with LlavA-Phi3 and *Q*_4_ being the option with highest success rate, reaching 97.5%. Despite the processing time increases for both VLMs, LlavA-Phi3 is almost three times faster than LlavA-7B. Both models do a reasonable, albeit not perfect, job in detecting PPE non-compliance. This method demonstrates that using more contextual prompts and searching for additional similar words produced by the VLM can improve system performance by relying solely on zero-shot approaches, rather than collecting data or training and fine-tuning models. This reduces time and effort while enabling the seamless development of intelligent safety systems. We do not claim that the system outperforms established baselines such as YOLO. Instead, we aim to highlight the potential of these VLMs, which, after careful prompt engineering, can achieve reasonably good accuracy without any data dependence. Additionally, in terms of response time, the observed latency of approximately 3 to 10 seconds is unlikely to materially affect safety outcomes, given that the monitored hazards typically evolve over substantially longer timescales. To this end, these timescales allow Chemist Eye to run offline models on low-resource computing equipment.

**Table 2:** Results for lab coat compliance detection for Llava:7b and Llava-phi3 models in terms of accuracy, hallucinations (Hall.) and time

Query	LLaVA-7B			LLaVA-Phi3		
	Accuracy (%)	Hall. (%)	Time (s)	Accuracy (%)	Hall. (%)	Time (s)
*Q*_1_	67.5	0.0	3.75	74.0	0.0	2.75
*Q*_2_	71.5	0.0	3.95	71.0	1.0	3.05
*Q*_3_	**84.0**	0.0	8.25	95.0	0.0	3.00
*Q*_4_	83.0	0.0	9.52	**97.5**	0.5	3.65

#### 2.3.2 Case study 2: worker accident detection *via* posture recognition

The second experiment involved recording videos of a lab user kneeling or crawling (to simulate an accident or medical emergency) and storing them in ROS bag files to evaluate the VLMs' ability to detect potential accidents. The same image categorisation process used in the first case study was applied. We evaluated the VLMs using queries *Q*_5_–*Q*_10_, also listed in Table 1. A similar strategy to that used in Experiment 1 was employed: we analysed the VLM outputs and observed that specific keywords were used more frequently to describe particular postures or actions. The same metrics (accuracy and hallucination rate) were used to quantify performance in this experiment.

In a similar setup to the PPE compliance tests, the video streams from the Chemist Eye RGB-D Stations were used to identify situations that might indicate an accident or a medical emergency. The accuracy rates reflect how effectively Chemist Eye distinguished between standing postures and postures that are related to accidents or medical emergencies, such as individuals lying, sitting, or crawling on the floor. Table 3 summarises the results. LlaVA-Phi3 performed better by achieving a 97% accuracy for recognising potential accidents. For both models, using *Q*_10_ proved to be the most effective strategy to spot possible accidents. Once again, injecting more context into the prompts and searching for more similar words in the VLM's response proved to improve the system's performance in detecting accidents, without relying on the collection of large datasets or the training or fine-tuning of models. Additionally, the latency times of LLaVA-Phi3, which exhibited the highest accuracy, demonstrate that the response time is shorter than the timescale over which these accidents typically evolve, supporting the feasibility of the approach without specialised computing infrastructure.

**Table 3:** Comparison of LLaVA-7B and LLaVA-Phi3 across different queries in terms of accuracy, hallucinations (Hall.) and time

Query	LLaVA-7B			LLaVA-Phi3		
	Accuracy (%)	Hall. (%)	Time (s)	Accuracy (%)	Hall. (%)	Time (s)
*Q*_5_	59.0	1.0	3.44	78.0	7.5	4.70
*Q*_6_	68.0	0.0	2.19	50.0	93.0	8.90
*Q*_7_	80.0	18.0	4.47	90.5	0.0	2.10
*Q*_8_	59.0	8.5	4.70	77.0	6.5	3.40
*Q*_9_	73.5	41.0	9.70	87.5	9.0	5.70
*Q*_10_	**88.0**	3.5	13.4	**97.0**	3.5	6.70

#### 2.3.3 Case study 3: automated response to PPE non-compliance

For the third experiment, multiple video streams showing an individual both wearing and not wearing a lab coat were fed into Chemist Eye. A 10-minutes countdown was set to trigger the system's automatic notification *via* Slack when the worker had not complied with the PPE requirements by the end of the countdown.

**Fig. 8:**
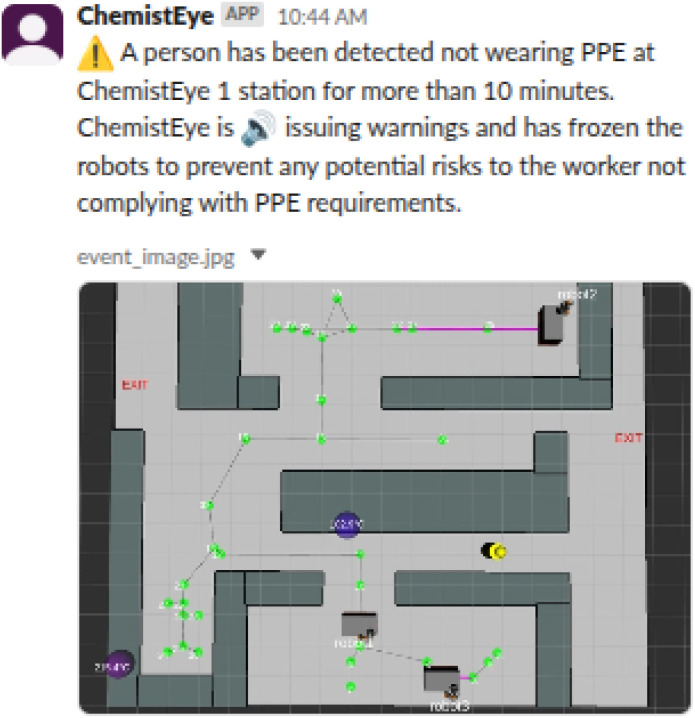
Chemist Eye notification of a worker not complying with PPE usage. If a worker has not complied with the PPE requirements by the end of the 10-minutes countdown, a notification is sent through Slack.

When Chemist Eye detects PPE non-compliance, it first freezes the mobile robots, reproduces several verbal alerts through the speakers of the closest Chemist Eye RGB-D station, and then triggers a countdown of 10 minutes; this parameter can be tuned, giving enough time for the individual to abide by the PPE rules. If 10 minutes pass and the system still detects PPE non-compliance, it then sends a notification through Slack to relevant personnel (see Fig. 8). We observed, due to the deterministic nature of this feature (being an if-else logic), that when the model detected the problem, Chemist Eye was 100% effective in preventing the robots from moving and notifying the issue once the countdown was over.

#### 2.3.4 Case study 4: worker accident response and robot repositioning

The fourth experiment involved randomly selecting locations for simulated worker accidents and then prompting two VLMs to determine the best navigation nodes for the robots to move to, based on the accident location. For this case study, we evaluated two VLMs: LLaVA-7B and LLaVA-Phi3. Each experiment required querying the models to suggest safe navigation nodes for three KUKA robots, using two map representations: a 2D schematic and a 3D RViz-style visualisation. The 2D prompts used symbolic representations (*e.g.*, triangles for people, orange squares for robots, red circles for fires), while the 3D prompts provided more realistic visuals (*e.g.*, meeples for people, URDF models for robots). Additionally, we tested the system under three prompting conditions: *c*_1_ (no list of nodes provided), *c*_2_ (full list of valid nodes included), and *c*_3_ (only a filtered list of safe nodes shown). Performance was evaluated using three error metrics: *e*_1_ (robots blocking each other), *e*_2_ (suggested nodes that do not exist), and *e*_3_ (robots positioned too close to the accident site).

Table 4 summarises the results for both models and both types of maps (2D and 3D). It can be observed that adding more context or structured information, such as the list of available nodes, as in the case of *c*_3_, improves the decision-making performance of both models. In particular, LLaVA-7B benefits significantly from filtered inputs, as does LLaVA-Phi3, achieving near-perfect success rates (*e.g.*, 10/10 in 2D *c*_2_, 9/10 in 3D *c*_3_), with an average of 95%. Furthermore, *e*_3_ (robot close to accident) is the most frequent error type across both models, with the *c*_1_ configuration being the most affected. This issue highlights the difficulty of spatial risk awareness when explicit contextual information is not provided to the models.

**Table 4:** Evaluation of decision-making by LLaVA-7B and LLaVA-Phi3 across 2D and 3D RViz map views

Map	Config	LLaVA-7B				LLaVA-Phi3			
		*e*_1_	*e*_2_	*e*_3_	Success rate	*e*_1_	*e*_2_	*e*_3_	Success rate
2D	*c*_1_	1	3	1	4/10	2	5	2	2/10
2D	*c*_2_	2	2	1	5/5	1	6	1	3/10
2D	*c*_3_	0	0	0	**10/10**	2	5	1	3/10
3D	*c*_1_	4	2	6	3/10	1	3	2	4/10
3D	*c*_2_	2	2	1	6/10	3	2	3	3/10
3D	*c*_3_	1	1	0	**9/10**	2	2	1	5/10

#### 2.3.5 Case study 5: fire detection and robot repositioning

We tested the system under three prompting conditions: *c*_1_ (no list of nodes provided), *c*_2_ (full list of valid nodes included), and *c*_3_ (only a filtered list of safe nodes shown). The filtered node list was generated by defining safety perimeters around people and risk areas. The prompts included a description of all relevant map elements—robots, fire markers, available nodes, and any additional visual features. Both VLMs were instructed to reply in a specific format (*e.g.*, ROBOT1: [*X*], ROBOT2: [*Y*], ROBOT3: [*Z*]), where 0 indicated no movement. Responses were parsed to extract the suggested node numbers for each robot. Performance was evaluated using three error metrics: *e*_1_ (robots blocking each other), *e*_2_ (suggested nodes that do not exist), and *e*_3_ (robots positioned too close to the accident site). For this case study, we also evaluated two VLMs: LLaVA-7B and LLaVA-Phi3.

**Fig. 9:**
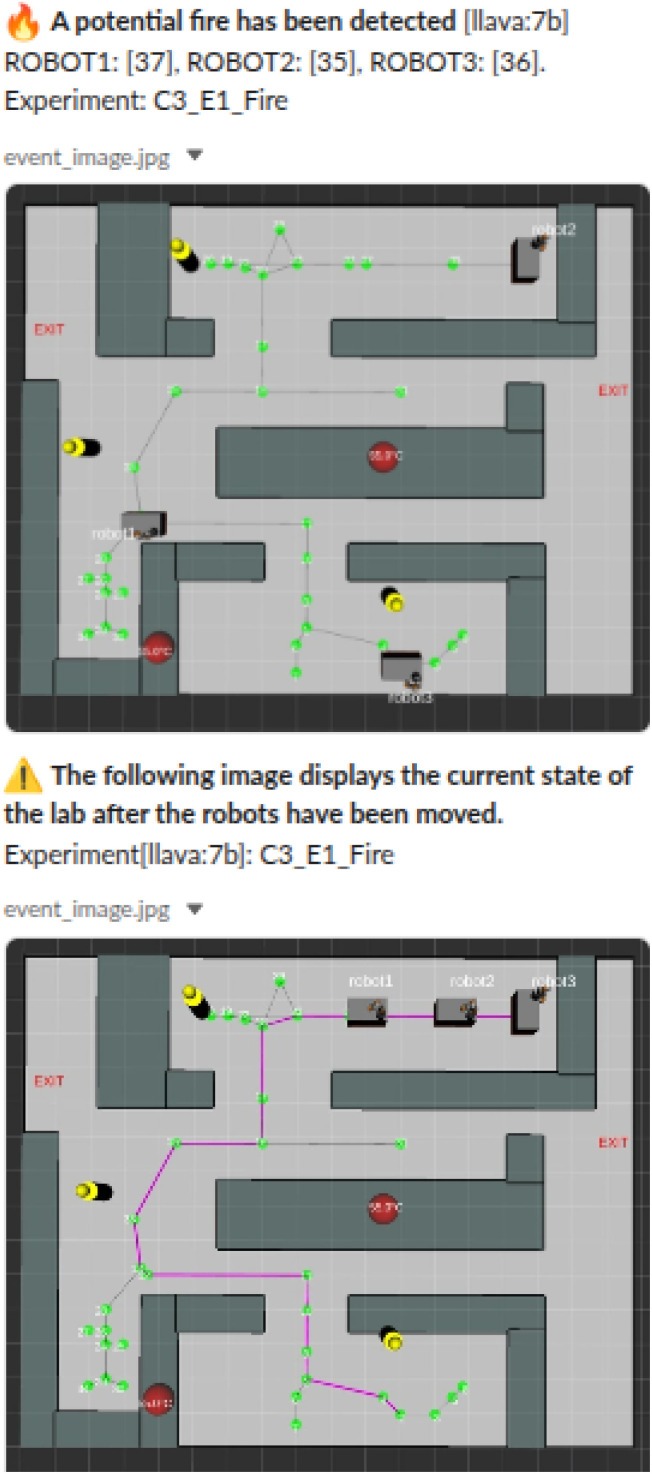
Chemist Eye notification about a potential accident.

Table 5 shows the performance of LLaVA-7B and LLaVA-Phi3 in fire detection scenarios across 2D and 3D RViz map views. Similar to the accident scenario, both models benefit from more contextual prompts. LLaVA-7B achieves a success rate of 9/10 in both views under configuration *c*_3_, while LLaVA-Phi3 reaches 10/10 in 3D, averaging a 95% of success rate, Fig. 9 shows a successful attempt of moving the robots away from the accident. Prompts not containing context (*c*_1_, *c*_2_) led to critical errors, particularly *e*_3_ (robot too close to accident). This behaviour highlights the importance of context injected into the query. Compared to the accident scenario, fire introduces more variability, making prompt clarity even more critical for safe robot navigation. In terms of latency for fire detection, the time for the IR camera station to transmit data to the ROS master averages 300 ms, which allows the system to detect a hazard within that time frame. However, in real scenarios, fires involving solvent spills can evolve within seconds. In such cases, the system may detect and report the hazard, but it is unlikely that the robots would be able to reach a safe position before the situation escalates. For the repositioning strategy, our aim is not to demonstrate that the VLM outperforms existing baselines or well-established robotic strategies. Instead, the value of the VLM lies in its ability to identify safe areas based on contextual information, such as the map layout. This represents an advance because it enables generalisation without relying on fixed rules, requiring only an input image and a contextual prompt. In contrast, classical approaches would struggle to adapt to changes in the map layout, whereas our method only requires the updated map and prompt.

**Table 5:** Evaluation of navigation node suggestions by LLaVA-7B and LLaVA-Phi3 in response to fire presence across 2D and 3D RViz map views

Map	Config	LLaVA-7B				LLaVA-Phi3			
		*e*_1_	*e*_2_	*e*_3_	Success rate	*e*_1_	*e*_2_	*e*_3_	Success rate
2D	*c*_1_	0	3	3	4/10	0	4	0	6/10
2D	*c*_2_	1	1	6	1/10	1	6	1	4/10
2D	*c*_3_	0	0	1	**9/10**	0	1	2	8/10
3D	*c*_1_	4	2	6	3/10	0	3	0	4/10
3D	*c*_2_	0	1	4	2/10	3	0	4	4/10
3D	*c*_3_	0	1	0	9/10	0	0	0	**10/10**

#### 2.3.6 Case study 6: extended evaluation

This case study aims to extend the evaluation of Chemist Eye by using a richer dataset containing multiple people and occlusions in the images, as described in the Methods subsection, as well as comparing the performance and differences between offline VLMs (*e.g.,* LLaVA), online VLMs (GPT-4o mini), and classical vision models (YOLO). Additionally, we evaluate the performance of GPT-4o mini to reduce the decision-making time observed with LLaVA-7B and LLaVA-Phi3.

First, we used the best-performing strategy for PPE detection from Case Study 1 (*Q*_4_) with LLaVA-7B and LLaVA-Phi3, and compared it with YOLOv8-pose. Since YOLOv8-pose can only detect keypoints corresponding to parts of the body, we implemented a strategy consisting of classifying an individual as prone when the torso angle relative to the horizontal is less than 45°, or the bounding box width exceeds its height, indicating that the person is lying on the floor. Additionally, to handle more than one individual in an image, the system crops the image corresponding to each individual and then analyses them separately, one by one. Table 6 summarises the results, where it can be observed that the average accuracy for detection is 86.07%, 90.33%, and 80.5% for LLaVA-7B, LLaVA-Phi3, and YOLOv8-Pose, respectively. The results show that YOLOv8-pose performed well in detecting individuals who are standing; however, for prone detection, it encountered difficulties. These difficulties originate from the dataset, which contains images of incomplete bodies and occlusions, making it more difficult to track keypoints. Since YOLOv8-pose lacks a mechanism to understand semantics, the VLMs showed a better overall understanding of the events occurring in the images.

**Table 6:** Prone detection: accuracy, hallucinations (Hall.) and time for LLaVA-7B, LLaVA-Phi3 and YOLO8n-Pose

Model	Prone (%)	Not prone (%)	Total (%)	Time (s)	Hall. (%)
LLaVA-7B	98.33	75.00	86.07	17.96	31.17
LLaVA-Phi3	97.07	83.00	90.33	12.00	19.50
YOLO8n-pose	63.33	97.67	80.50	0.009	n/a

Second, we used the best-performing strategy for detecting PPE compliance from Case Study 2 (*Q*_10_). LLaVA-7B and LLaVA-Phi3 correctly classified 86.07% and 82.83% (see Table 7), respectively, of the images in the dataset containing multiple users and occlusions. Regarding methodologies using YOLO for PPE classification, several studies focus on construction,^39^ achieving a performance of approximately 86.55%. However, these studies involve more object types (helmet, glasses, gloves, vest, *etc.*) than those included here, and occlusions make it more challenging for YOLO to perform better. Moreover, datasets containing users wearing PPE in a chemistry automation laboratory are scarce. For example, this dataset^40^ contains only 250 images of a single user, with or without a lab coat and glasses, in a laboratory setting. To compare YOLO fairly with our approach, it would be necessary to collect a sufficiently large dataset (at least 1000 images per class), define classes in YOLO (*e.g.*, lab coat, glasses, gloves), and train a model. This would contradict the motivation of this paper, which is to leverage an already trained VLM, improve contextual prompts, and avoid collecting data to achieve reliable performance.

**Table 7:** PPE detection: accuracy, hallucinations (Hall.) and time for LLaVA-7B and LLaVA-Phi3

Model	PPE (%)	No PPE (%)	Total (%)	Time (s)	Hall. (%)
LLaVA-7B	90.67	86.00	86.07	12.00	0.0
LLaVA-Phi3	89.00	76.67	82.83	5.82	0.0

Third, to reduce decision-making times, we used GPT4o-mini, an online model, together with the best-performing strategy from Case Studies 5 and 6. This strategy consists of feeding the model with *c*_3_ (a filtered list of safe nodes), an explanation of the context, and a 2D or 3D image of the RVIZ map. Tables 8 and 9 summarise the results. It can be observed that the success rate is similar to that achieved by LLaVA models in Case Studies 4 and 5, approximately 95%. In terms of time, the average API response is 2.4 seconds, representing an improvement; however, this approach makes the system dependent on an internet connection, increases operational costs, and subjects it to the privacy policies of a third-party service.

**Table 8:** Worker accident reaction times and success per trial using GPT-4o mini. Strategy: *C*_3_. Times in seconds

Trial	2D *C*_3_		3D *C*_3_	
	Time (s)	Success	Time (s)	Success
1	3.106	✓	2.193	✓
2	2.800	✓	2.490	✓
3	2.600	✓	2.258	✗
4	1.600	✓	2.141	✓
5	2.550	✓	1.700	✓
6	2.800	✓	2.198	✓
7	2.300	✓	1.820	✓
8	2.660	✓	1.590	✓
9	2.130	✓	2.118	✓
10	2.200	✓	1.527	✓
Avg.	2.470	10/10	2.635	9/10

**Table 9:** Fire reaction times and success per trial using GPT-4o mini. Strategy: *C*_3_. Times in seconds

Trial	2D *C*_3_		3D *C*_3_	
	Time (s)	Success	Time (s)	Success
1	1.799	✓	3.570	✓
2	1.871	✓	2.569	✓
3	1.782	✓	2.856	✓
4	2.940	✓	3.077	✓
5	2.138	✓	1.901	✓
6	1.973	✓	2.049	✗
7	1.846	✓	2.505	✓
8	1.742	✓	2.411	✓
9	2.917	✓	2.035	✓
10	1.841	✓	1.863	✓
Avg.	2.080	10/10	2.281	9/10

Lastly, Table 10 presents a comparison of the different models used in this study. Overall, YOLO is a cost-effective and fast solution when a dataset is available to train new classes. The community has reported several successful use cases in which large datasets were produced in parallel, benefiting multiple areas of expertise. In the context of laboratory automation, a VLM is a better alternative when processing time is not critical and creating datasets is challenging due to safety and privacy constraints inherent to the environment. Additionally, the reasoning capabilities of VLMs can be leveraged not only for classification and decision-making but also for broader activities, such as experiment monitoring or human–laboratory interaction. The architecture of Chemist Eye allows these technologies to be combined, providing users with options to optimise the system according to their specific needs.

**Table 10:** Comparison between traditional computer vision (YOLO), offline VLMs (*e.g.*, LLaVA), and online VLMs (*e.g.*, GPT-4o mini) for laboratory safety monitoring

Capability	YOLO	Offline VLM	Online VLM
PPE detection	✓	✓	✓
Pose detection	✓	✓	✓
Accident reasoning	✗	✓	✓
Robot decision-making	✗	✓	✓
Zero-shot adaptation	✗	✓	✓
Multi-task reasoning	Limited	✓	✓
Task generalisation	✗	✓	✓
Offline operation	✓	✓	✗
Online requirement	✗	Optional	✓
Privacy	High	High	Depends on provider
Cost	Low	Low	Medium–High
Speed	Very fast	Moderate	Fast (low latency APIs)
Performance	High	High	High

### 2.4 Discussion

Chemist Eye integrates a range of technologies to control robots, monitor SDL conditions in real-time, and notify users about potential accidents. Moreover, using VLMs for detecting PPE compliance and accidents related to workers proved to be reliable, at least in the cases presented herein, and the models achieved reasonable accuracy without any modifications. Traditional supervised detectors such as YOLO typically require task-specific datasets and retraining, however for these VLMs, this data collection and training was not necessary, saving a lot of time and accelerating the development of Chemist Eye. However, there are distinct limitations in terms of zero-shot decision-making; for example, the two models came across significant challenges, demonstrating the need for more context feeding in the query to achieve reasonable performance. In this context, a key insight from this work is that structured contextual prompting can substantially improve VLM reliability without requiring task-specific fine-tuning or curated training datasets. This is particularly valuable in academic laboratory environments where large-scale image collection raises ethical, privacy, and regulatory challenges.

Indeed, in this first version of Chemist Eye, the decision-making failed most of the time when not providing enough contextual information in the query and even repositioned robots close to a potential fire, something a human would definitely avoid by only looking at the map without the need for further context or explanations. This shows clearly that these VLMs are not yet trustworthy for making autonomous safety-related decisions, although they do show real promise for issuing alerts to human users who can then make appropriate context-based decisions. In terms of latency, while 3–10 seconds is acceptable for detecting a prone individual, and 0.3 seconds is sufficient for detecting fires, in rapidly evolving situations, the system may notify about the hazard but might not be able to reposition the robots in time. Additionally, in extreme scenarios such as solvent fires or explosions, hazard escalation may occur faster than the time required for robot relocation. In such cases, the primary role of the system should be early detection and human notification rather than attempting autonomous robot repositioning.

Hence, future improvements could focus on the decision-making model by incorporating additional spatial awareness constraints. Additionally, defining predefined ‘safe areas’ for robots (*i.e.*, a zone that is well away from any possible sources of fire and away from any lab exits or entrances) could simplify the heuristics and the decision-making, although even here there are considerations such as determining the shortest and safest route to that ‘safe zone’, avoiding the detected hazard. Despite the challenges faced, this work suggests that safety intelligence may no longer require large supervised datasets in the future, lowering the barrier for deploying protective monitoring in emerging autonomous laboratories. Chemist Eye is a system designed to complement existing laboratory safety infrastructure rather than replace it. Fire alarms, smoke detectors, fume hood fire suppression systems, emergency showers, and emergency shutoff controls remain the primary safety measures. Our system adds an additional monitoring layer capable of detecting risks and controlling laboratory robots if necessary, capabilities that a traditional safety system cannot provide.

## 3 Conclusions

In this paper, we have introduced and validated Chemist Eye through experiments involving real-world scenarios and data. The system demonstrated the potential to identify accidents and PPE non-compliance, and it uses this information for decision-making. Future work could extend the model to identify whether a user is wearing safety glasses and gloves, but these checks require further steps due to occlusions that may lead the VLM to misinterpret the camera stream and trigger false alarms or false positives; for example, standard glasses could be confused for safety glasses. While Chemist Eye has limitations and is not yet ready for full-scale use as a safety system, it is the first implementation of its kind for SDLs. Future validation under tightly controlled safety supervision could further assess system behaviour. Prior to this, simulation-based benchmarking remains the appropriate first step for safety-critical laboratory environments. The benefits for autonomous laboratory safety provided by systems such as Chemist Eye might influence and shape the design of SDLs in the future leveraging AI-driven reasoning and decision-making. Beyond the concepts presented here, the development of systems that enable a more adaptive safety supervision in places where humans and robots coexist will contribute to safer working environments. While there are significant pitfalls in relying on AI for safety, and we would never advocate replacing human judgment, we believe that systems such as Chemist Eye, with extensive testing and benchmarking, might help to create safer laboratories in the future.

## Author contributions

Funding acquisition and resources (AC). Project administration and supervision (AC, HF). Methodology (FMG, ZZ, SV, LL, RC and AC). Formal analysis and investigation (FMG, AC). Conceptualisation, data curation, validation, writing – original draft and software (FMG). Writing – review & editing (FMG, AC, LL, HF and ZZ).

## Conflicts of interest

There are no conflicts to declare.

## Data Availability

The ROS package and software implementation of Chemist Eye used in this study are openly available at: https://doi.org/10.5281/zenodo.19508611 CAD designs, ROS bag files and datasets for reproducing the custom hardware and experiments are available *via* Zenodo: https://doi.org/10.5281/zenodo.19206709.
